# Thoracoscopic excision of two bronchogenic cysts located in highest upper mediastinum: Report of two cases

**DOI:** 10.12669/pjms.313.6921

**Published:** 2015

**Authors:** Fengwu Lin, Chuan Zhang, Kunpeng Cheng, Dan Dang, Yan Zhao

**Affiliations:** 1 Fengwu Lin, Department of Thoracic Surgery, China-Japan Union Hospital of Jilin University, Changchun 130033, China; 2 Chuan Zhang, Department of Pediatric Surgery, The First Hospital of Jilin University, Changchun 130021, China; 3 Kunpeng Cheng, Department of Thoracic Surgery, China-Japan Union Hospital of Jilin University, Changchun 130033, China; 4 Yan Zhao, Department of Endocrine, The Second Hospital of Jilin University, Changchun 130041, China

**Keywords:** Brochogenic cysts, Highest upper mediastinum, Thoracoscopy

## Abstract

Bronchogenic cysts are rare congenital malformation that need surgical removal. To date, bronchogenic cysts located in highest upper mediastinum excised by thoracoscopy have not been reported, though complete thoracoscopic extirpation of a bronchogenic cyst has been reported before. We excised two highest upper bronchogenic cysts by thoracoscopy successfully without any postoperative complication, demonstrating thoracoscopy could be a first-line therapeutic option even for highest upper mediastinum brochogenic cysts.

## INTRODUCTION

Bronchogenic cysts are uncommon congenital cystic lesions that need surgical excision. Open operation has been increasingly replaced by thoracoscopic excision of bronchogenic cysts,^1^-^3^ which are recommended to be removed for pathologic diagnosis, symptom relief, and to prevent complications.^4^ However, there are no reports found in the literature that highest upper mediastinal cysts were resected by thoracoscopy so far, probably due to the hidden and complex anatomical relationship of the highest mediastinal brochogenic cysts. The highest upper mediastinal cyst is defined as cysts that are located in superior mediastinum, where anatomical relations of cysts are complex.

To date, we have successfully conducted two cases of thoracoscopic excision of highest upper mediastinal cysts, and noticed some findings concerning thoracoscopic resection of highest mediastinal cysts.

## CASE REPORT

During 2013, two patients with mediastinal bronchogenic cyst underwent the thoracoscopy in our hospital. Both patients were female, one 46-year-old while another 62-year-old, complaining of mild pain and discomfort in the upper chest or the root of neck, accompanied by paroxysmal cough but no mucus, and without positive signs. Contrast enhanced computer tomography (CT) was performed in both patients, displaying an unilocular cystic lesion about 2.0×2.0 cm in size far above the aortic arch and close to the cupula pleurae in one patient, and two lesions above the cupula pleurae in the other patient. Subsequent fiberoptic bronchoscopy revealed no abnormal condition.

**Fig.1:**
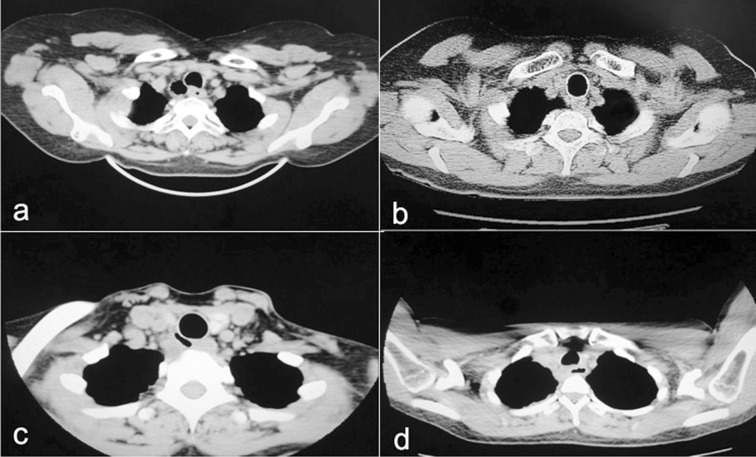
Preoperative pictures of two patients (a, c) versus accordingly postoperative pictures (b, d).

Both patients were incubated with a double-lumen endotracheal tube, and placed in the lateral decubitus position. The cameral port was placed in the 7^th^ intercostal space across the posterior axillary line, and the two operating ports were placed respectively in the 4^th^ intercostal space across the anterior axillary line and the 8^th^ intercostal space across the angulus inferior scapulae line. The masses were soft, smooth, and encapsulated, with complex anatomic relationship to adjacent tissues. Be careful to completely dissect away cysts with electrocautery or aspirator, and nourishing vessels were treated with electric coagulation or titanium clips. The operations went smoothly without conversion to thoracotomy. Chest tubes were removed respectively on postoperative day 3 or 5. Length of postoperative hospital stay was respectively 5 or 7 days. Both histological examinations proved congenital brochogenic cysts. Preoperative and postoperative CT images were shown as below (Fig.1).

## DISCUSSION

Bronchogenic cysts manifest as solitary or multiple lesions, majority of which are located in the mediastinum while others occur in the lung parengchyma.^5^ Once a bronchogenic cyst has been diagnosed radiologically, it should be excised to establish the histopathological diagnosis, prevent complications including infection, hemoptysis, rupture, or fear of malignancy.^6^

Usually thoracotomy enters the thoracic cavity through the 3^th^ intercostal space, and cuts part of latissimus dorsi, serratus anterior muscle, and trapezius muscle, which cause patients great damage. Even so, the disadvantages of thoracotomy are apparent including poor access, limited surgical fields and more required practical experience in this procedure, lack of satisfactory visions of crervical pleura abutting root of neck. In contrast, thoracoscopic excision of bronchogenic cysts has such evident merits as less traumas, rapid recuperation and more beautiful incisions.^3^ Since the first case reported in the literature in 1991,^7^ a series of similar cases were reported demonstrating less postoperative pain, decreased chest tube duration, and shorter hospital stay with thoracoscopic surgery.^8^ However none of them concerned so high bronchogenic cyst near to the root of neck excised by thoracoscopy.

We summarized the reasons for great difficulty in thoracoscopic excision of bronchogenic cysts located in highest upper mediastinum as below. Firstly, the anatomic relations of upper mediastinal bronchogenic cysts were very complex, where the subclavian artery and vein are around. In addition, the cyst wall is typically thick, tightly adhesive to the trachea so that the dissection of cyst is difficult. Finally, the cystic lesions may be so small and hidden in the upper mediastinum that it is hard to detect them, especially in video-assisted thoracoscopy.

As regard these features of highest upper mediastinal bronchogenic cysts, we recommended some corresponding countermeasures. Preoperative chest CT or contrast-enhanced CT was performed to determine the anatomic relations of the cystic lesion with surrounding tissues. CT can give information on the size and shape of the cyst, and its anatomic relations with surrounding vital structures.^9^ In case the lesion was very small and hidden in the cupula of pleura, it is necessary to cut open pleura mediastinalis. Also, the cystic wall may be relatively thick and tough, indistinguishable from the subclavian artery. Especially in elderly patients, the subclavian artery may be cut off by mistake due to arteriectasia. Cystic lesions frequently localize behind the subclavian artery. Blunt dissection by endoscopic hemostatic forceps is recommended between the cyst and subclavian arterty, and take care of recurrent laryngeal nerve. In the process of dissecting the cyst, loose adhesion of the cyst away from the bronchus should be dealt with first, and the relatively tight adhesion of the lesion with the bronchus should be treated in the end. To avoid the injury of bronchus, blunt dissection can be carried out on the side near the cyst. Even if the cyst is ruptured and cannot be removed completely, the germinal layer should be scraped and treated with carbolic acid or electric cauterization.^10^ Nevertheless, thoracoscopic surgery for bronchogenic cysts has some limitations. Since bronchi are similar to blood vessels in appearance, surgeons cannot directly touch and feel the tissue under thoracoscope. In case of the rupture of a blood vessel or bronchus, conversion to thoracotomy may be needed.

Compared to conventional thoracotomy, thoracoscopic resection of bronchogenic cysts has obvious advantages including less injury, rapid recovery, and much clearer operation vision of the lesion. Our practice has proved that thoracoscopic excision of highest upper mediastinal bronchogeinc cysts is an alternative of decided advantages.
